# Orbital Magnetic Moment Controlled Converse Magnetoelectric Effect in bcc‐Co_3_Mn/Fe/V/PMN‐PT Multiferroic Heterostructures

**DOI:** 10.1002/advs.202522581

**Published:** 2026-01-27

**Authors:** Takamasa Usami, Yuichi Murakami, Ryota Watarai, Yu Shiratsuchi, Yoshihiro Gohda, Kohei Hamaya

**Affiliations:** ^1^ Spintronics Research Network Division, Institute for Open and Transdisciplinary Research Initiatives The University of Osaka Suita Osaka Japan; ^2^ Center for Spintronics Research Network, Graduate School of Engineering Science The University of Osaka Toyonaka Osaka Japan; ^3^ Department of Systems Innovation, Graduate School of Engineering Science The University of Osaka Toyonaka Osaka Japan; ^4^ Department of Materials Science and Engineering Institute of Science Tokyo Yokohama Kanagawa Japan; ^5^ Department of Materials Science and Engineering, Graduate School of Engineering The University of Osaka Suita Osaka Japan

**Keywords:** bcc Co_3_Mn, converse magnetoelectric effect, multiferroic heterostructures, PMN‐PT

## Abstract

To demonstrate multiferroic heterostructures with a giant converse magnetoelectric (CME) effect, we propose a material design strategy based on utilizing the control of orbital magnetic moments. Following this strategy, we focus on a multiferroic heterostructure composed of (211)‐oriented metastable body‐centered cubic (bcc) ferromagnetic Co_3_Mn, which is predicted to exhibit a significant change in magnetic anisotropy due to in‐plane piezostrain. By inserting an appropriate Fe/V layer, we experimentally obtain a highly (211)‐oriented bcc Co_3_Mn layer on piezoelectric Pb(Mg_1/3_Nb_2/3_)O_3_‐PbTiO_3_(011). Using this structure, we reproducibly show a giant CME effect with repeatable and nonvolatile magnetization vector switching. Our proposal is one of the important strategies toward the development of room‐temperature electric‐field‐controlled spintronic devices with ultra‐low power consumption.

## Graphical abstract

Based on orbital magnetic moment control, a material design strategy is proposed for a giant converse magnetoelectric effect in multiferroic heterostructures. This study will pioneer a promising route toward low‐power spintronic devices with an electric field.

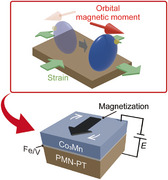

## 1 Introduction

Magnetoelectric random‐access memory (MeRAM) has attracted attention as a promising next‐generation magnetoresistive RAM (MRAM) concept due to its low energy consumption during data writing [1, 2]. The key component for low‐energy data writing is a multiferroic heterostructure consisting of ferromagnetic (FM) and piezoelectric layers. Applying an electric field (*E*) to the multiferroic heterostructure induces strain transfer from the piezoelectric layer to the FM layer, resulting in a change in magnetic properties through a strain‐mediated converse magnetoelectric (CME) effect [2, 3, 4, 5, 6, 7, 8]. This enables *E*‐control of the magnetization vector of the free layer in the magnetic tunnel junctions in MeRAM [1, 2].

**FIGURE 1:**
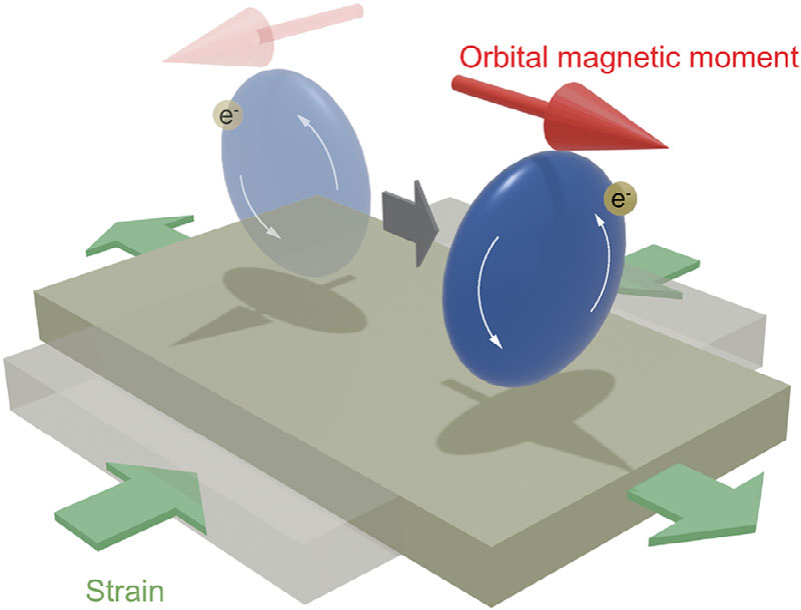
Schematic of orbital moment anisotropy control in the ferromagnetic layer of the multiferroic heterostructure. The blue oblate (pancake‐type) spheroid represents an electron's distribution in a ferromagnetic material, and the red arrows indicate the resulting orbital magnetic moments. The green arrows indicate the strain induced by the piezoelectric layer.

The modification of the magnetic easy axis through the CME effect can be understood in terms of a competition between the magnetic anisotropy energy induced in the FM layer and the magnetoelastic energy arising from the piezoelectric layer, as pointed out by Wang et al. [9]. Basically, the former magnetic anisotropy energy can be treated as a sum of two energy terms. One is associated with the anisotropy of orbital magnetic moments (Δ*m*_orb_), and the other is linked to the magnetic moment as a result of the quadrupole moment of the electron density distribution (*m*_D_). In general, the magnetic anisotropy energy (*E*_MA_) is expressed in the following equations [10, 11, 12, 13, 14].

(1) $$E_{\mathrm{MA}}=-\frac{1}{4\mu_{B}}\sum \xi \Delta m_{\mathrm{orb}}+\frac{3}{8\mu_{B}}\sum \frac{\xi^{2}}{\Delta_{\mathrm{exc}}}m_{D}$$

where the summations are made for atomic sites and orbitals. Here, *μ*_B_, *ξ*, and Δ_exc_ are the Bohr magneton, the spin‐orbit coupling constant, and the averaged exchange splitting energy, respectively. Therefore, if the electron's occupation of appropriate materials is intentionally modified via strain as schematically shown in Figure 1, we can efficiently change Δ*m*_orb_ in the first term, leading to the artificial control of the CME effect through tuning the magnetic‐anisotropy energy.

Very recently, we have demonstrated that the multiferroic heterostructure consisting of Co_2_FeSi and Pb(Mg_1/3_Nb_2/3_)O_3_‐PbTiO_3_ (PMN‐PT) shows a giant CME effect [15, 16, 17]. By introducing a vanadium (V) insertion layer, we achieved a highly oriented Co_2_FeSi layer, enabling anisotropic control of *m*_orb_ via in‐plane piezostrain. As a result, piezostrain‐induced control of the magnetic anisotropy was achieved artificially [17], and a subsequent study demonstrated electric‐field control of the TMR ratio by implementing this heterostructure into magnetic tunnel junctions (MTJs) [18]. Thus, if this strategy for controlling the CME effect can be applied to other multiferroic heterostructures, we can expand the material selection for MeRAMs.

As an appropriate FM material to verify the strategy previously mentioned, we focus on a metastable body‐centered cubic (bcc) Co_3_Mn alloy among several FM materials because there are many studies of FM electrodes for MTJ devices [19, 20, 21, 22, 23, 24]. Furthermore, a large TMR ratio over 200% at room temperature was achieved in magnetic tunnel junctions incorporating bcc Co_3_Mn electrodes [20, 21, 22]. Concentrating on the fascinating features of Co_3_Mn, we previously fabricated a multiferroic heterostructure consisting of metastable bcc Co_3_Mn on piezoelectric PMN‐PT using an molecular beam epitaxy (MBE) technique [25, 26]. The CME effect was demonstrated in the Co_3_Mn/Fe/PMN‐PT(001) heterostructures, and the value of the CME coupling coefficient *α*_E_ was approximately 8.3 × 10^−6^ s m^−1^ [25, 26]. If we can use anisotropic control of *m*_orb_ via in‐plane piezostrain in the the Co_3_Mn‐based multiferroic heterostructures, the large CME effect can be expected.

## 2 Results

### 2.1 Prediction of Δ*m*_orb_ Under the In‐Plane Strain

Since the CME effect of multiferroic heterostructures is strongly related to the strain‐induced modulation of magnetocrystalline anisotropy (MCA) [15, 16], we first perform first‐principles calculations to examine the MCA energy (*E*_MCA_) and to compare the strain‐induced *E*_MCA_ between (211)‐oriented and (001)‐oriented bcc Co_3_Mn. Here, two unit cells were used to calculate (211)‐oriented and (001)‐oriented Co_3_Mn. While the conventional cubic unit cell was used with the *a* vector along the [100] direction and the *b* vector along the [010] direction for (001)‐oriented Co_3_Mn, the orthorhombic unit cell was defined with the *a* vector along the [0$\bar{1}$1] direction and the *b* vector along the [1$\bar{1}\bar{1}$] direction for (211)‐oriented Co_3_Mn. For each case, the lattice constant *a* is strained from its equilibrium value *a*_0_, and the *b* direction is deformed from the equilibrium lattice constant *b*_0_ with the constraint *ab* = *a*_0_ *b*_0_. Under these constraints, the length of the *c* axis is optimized to minimize the total energy. In this study, *E*_MCA_ is defined as *E* ^*b*^ − *E* ^*a*^, where *E* ^*a*^ (*E* ^*b*^) is the total energy per atom with the magnetization in the *a* (*b*) direction. Δ*m*_orb_ is defined as $m_{\mathrm{orb}}^{b}-m_{\mathrm{orb}}^{a}$, where the orbital moments $m_{\mathrm{orb}}^{a}$ and $m_{\mathrm{orb}}^{b}$ are defined in the same fashion and averaged per atom.

**FIGURE 2:**
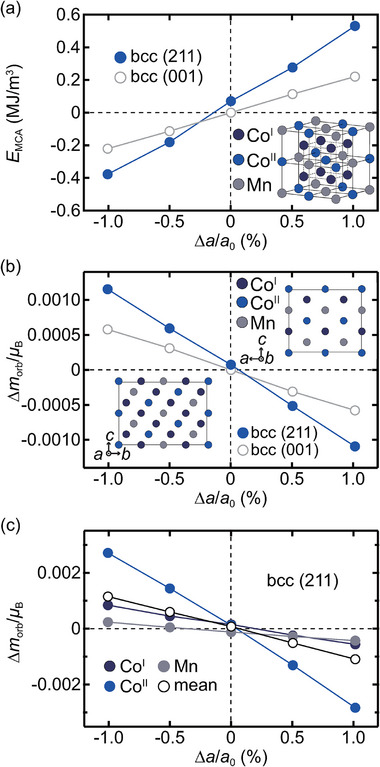
(a) The magnetocrystalline anisotropy (MCA) energy (*E*_MCA_) and (b) the orbital‐moment anisotropy (Δ*m*_orb_) of Co_3_Mn under the in‐plane strain as a function of Δ*a*/*a*_0_ = (*a* − *a*_0_)/*a*_0_ calculated from first principles. Here, the calculations regarding (001)‐oriented bcc Co_3_Mn are referred from our previous study [26]. The insets schematically show the unit cells of (a) (001)‐oriented Co_3_Mn and (b) (211)‐oriented Co_3_Mn. (c) Element‐specific Δ*m*_orb_ per atom under the in‐plane strain regarding (211)‐oriented bcc Co_3_Mn.

The calculated *E*_MCA_ for (211)‐oriented and (001)‐oriented Co_3_Mn are shown in Figure 2a. The results indicate that the strain‐induced *E*_MCA_ for (211)‐oriented Co_3_Mn is relatively large compared to that for (001)‐oriented Co_3_Mn, theoretically suggesting that (211)‐oriented Co_3_Mn is promising for achieving a large CME effect. We also calculate the mean values of Δ*m*_orb_ for both (211)‐oriented and (001)‐oriented Co_3_Mn, as shown in Figure 2b. Comparing the strain dependence of *E*_MCA_ and Δ*m*_orb_, we find that *E*_MCA_ is nearly proportional to Δ*m*_orb_.

We also examine the element‐specific Δ*m*_orb_ per atom for (211)‐oriented Co_3_Mn under in‐plane strain, as shown in Figure 2c. Here, Co^I^ and Co^II^ indicate Co atoms at the 8*c* and 4*b* sites, respectively, as shown in the insets of Figure 2a,b. Δ*m*_orb_ of all elements decreases as Δ*a*/*a*_0_ increases. In particular, Δ*m*_orb_ for Co^II^ shows a significant feature indicating a marked effect on the magnitude of the mean Δ*m*_orb_, leading to enhancing the strain‐induced *E*_MCA_. This feature is in contrast to the case of (001)‐oriented bcc‐Co_3_Mn [26], where the strain dependence of Δ*m*_orb_ varies depending on the atomic sites: Δ*m*_orb_ of the Co^I^ decreases as Δ*a*/*a*_0_ increases, while that of the Co^II^ increases with increasing Δ*a*/*a*_0_ [26]. Therefore, these results show that consideration of the crystallographic orientation of bcc‐Co_3_Mn is important for achieving a large strain‐induced *E*_MCA_.

### 2.2 Growth of bcc Co_3_Mn and Structural Characterization

To fabricate multiferroic heterostructures consisting of a (211)‐oriented Co_3_Mn layer, we employ an MBE technique, where the experimental details are provided in the Methods section. Here, to achieve metastable bcc (211)‐oriented Co_3_Mn on PMN‐PT, we focus on the use of Fe/V bilayer buffers. Here, the Fe thin layer between Co_3_Mn and PMN‐PT promotes the formation of the metastable bcc structure of the Co_3_Mn layer [25, 26], and the V layer on PMN‐PT(011) efficiently promotes the crystal growth of the film grown on it [17]. Thus, we utilize both advantages of the Fe/V bilayer buffer for the growth of (211)‐oriented Co_3_Mn on PMN‐PT(011).

**FIGURE 3:**
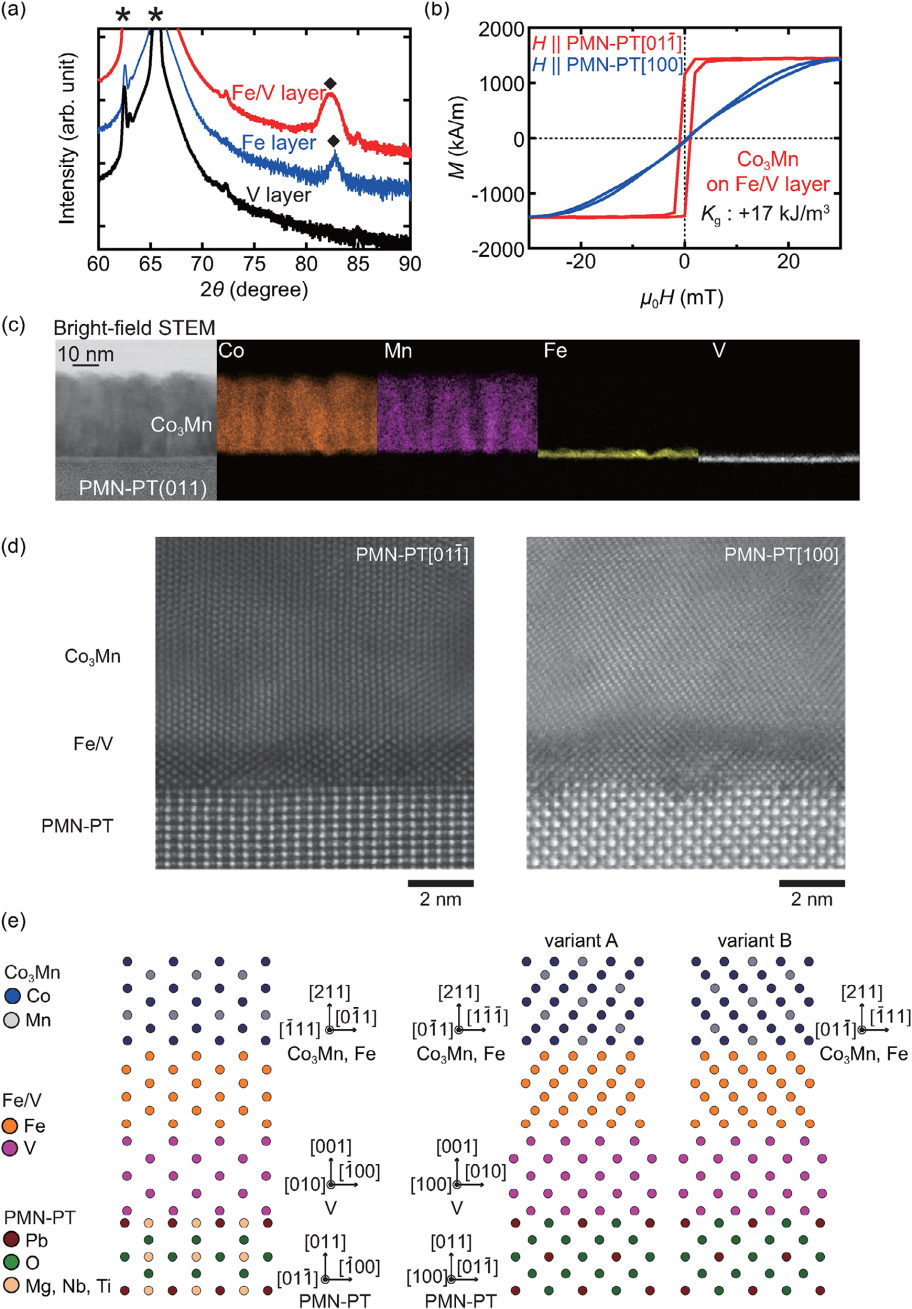
(a) X‐ray diffraction (XRD) *ω*−2*θ* profiles for the Co_3_Mn/Fe/V/PMN‐PT(011) heterostructure (red), Co_3_Mn/Fe/PMN‐PT(011) heterostructure (blue), and Co_3_Mn/V/PMN‐PT(011) heterostructure (black). The peaks denoted by the black diamond and asterisks originate from the Co_3_Mn(211) and PMN‐PT substrate, respectively. (b) Magnetization curves for the Co_3_Mn with Fe/V layers, where the external magnetic fields (*H*) are applied along the PMN‐PT[01$\bar{1}$] (red) and PMN‐PT[100] (blue) crystallographic directions. (c) Bright‐field cross‐sectional STEM images and EDX elemental maps of Co_3_Mn/Fe/V/PMN‐PT(011) heterostructure with zone axis of PMN‐PT[100]. (d) Enlarged HAADF‐STEM images for the Co_3_Mn/Fe/V/PMN‐PT(011) heterostructure with zone axes of PMN‐PT[01$\bar{1}$] (left) and PMN‐PT[100] (right). (e) Schematic illustrations of possible atomic arrangements determined from the HAADF‐STEM images with zone axes of PMN‐PT[01$\bar{1}$] (left) and PMN‐PT[100] (right).

Figure 3a shows X‐ray diffraction (XRD) *ω*‐2*θ* patterns for a Co_3_Mn(5)/Fe(2)/V(2)/PMN‐PT(011) heterostructure, together with a Co_3_Mn(5)/Fe(2)/PMN‐PT(011) and a Co_3_Mn(5)/V(2)/PMN‐PT(011) heterostructures (thickness is in nm). When only the V layer was used as a buffer layer, there is almost no diffraction peak at approximately 82°, indicating the absence of the (211)‐oriented Co_3_Mn. The profile for using the Fe(2)/V(2) buffer layer contains a distinct diffraction peak corresponding to bcc Co_3_Mn(211) at approximately 82°, confirming that the Co_3_Mn layer has a bcc structure. Also, a relatively small diffraction peak at approximately 82° is seen in the profile for using the Fe(2) buffer layer. Note that in the wide range scan shown in Figure S1, only the Co_3_Mn 211 diffraction peak is detected, and other diffraction peaks due to Co_3_Mn are not observed for the heterostructures using Fe(2)/V(2) and Fe(2) buffer layers. Based on these results, we conclude that using Fe layer plays a role in obtaining bcc structure. In addition, inserting the V layer between Fe and PMN‐PT enhances the growth of the (211) orientation of Co_3_Mn layers on top of PMN‐PT(011).

Figure 3b shows the magnetic‐field dependence of in‐plane magnetization for the Co_3_Mn on Fe/V, where the external magnetic fields (*H*) are applied along the PMN‐PT[01$\bar{1}$] (red) and PMN‐PT[100] (blue) crystallographic directions, measured by using a vibrating sample magnetometer (VSM) at room temperature. We note that clear in‐plane magnetic anisotropy is seen for the Co_3_Mn layer on the Fe/V layer, but the feature is markedly different from those of the Co_3_Mn grown on the Fe layer [Figure S2b] and the V layer [Figure S3a]. To quantitatively estimate the magnetic anisotropy (*K*_g_), we use the following relationship,

(2) $$K_{g}=\int_{0}^{M_{s}}\left(H_{100}-H_{01\bar{1}}\right)dM$$

where *M*_s_, *H*_100_, and $H_{01\bar{1}}$ are the saturation magnetization, the applied magnetic field along PMN‐PT[100], and that along PMN‐PT[$01\bar{1}$], respectively. The value of *K*_g_ for the Co_3_Mn on the Fe/V layer is ∼17 kJ m^−3^, larger than that for the Co_3_Mn on the only Fe (*K*_g_ ∼ 2 kJ m^−3^) shown in Figure S2b. Here, as shown in Figure S3a, the opposite magnetic anisotropy is evaluated (*K*_g_ ∼ −3 kJ m^−3^), for the Co_3_Mn on the only V layer. From these results, we can infer that the (211)‐orientation of Co_3_Mn layer is strongly related to the enhancement in *K*_g_ that can be modulated by Δ*m*_orb_ in bcc Co_3_Mn, as expected in Figure 2.

For the detailed structural characterizations, we observe cross‐sectional scanning transmission electron microscope (STEM) images. Figure 3c shows the bright‐field cross‐sectional STEM image and energy dispersive X‐ray spectroscopy (EDX) elemental maps of the Co_3_Mn/Fe/V/PMN‐PT(011) heterostructure. The thickness of the Co_3_Mn layer for the structural characterization is set to 30 nm. The EDX elemental map indicates that the V layer is uniformly grown on the PMN‐PT(011) substrate. Furthermore, owing to the uniform V layer, the Fe layer exhibits relatively small roughness, compared to the heterostructure with only the Fe insertion layer in Figure S2a. Atomic force microscopy (AFM) also revealed that the root mean square (RMS) surface roughness (*R*_RMS_) decreases from 1.2 to 0.78 nm by inserting the V layer. Therefore, these results suggest that the combination of the Fe and V layers is effective for obtaining a bcc Co_3_Mn layer with reduced roughness on PMN‐PT(011). This achievement is significant for integrating multiferroic heterostructures into MTJ devices.

Figure 3d shows enlarged high‐angle annular dark field (HAADF)‐STEM images for the Co_3_Mn/Fe/V/PMN‐PT(011) heterostructure with zone axes of PMN‐PT[01$\bar{1}$] (left) and PMN‐PT[100] (right). A sharp interface is observed in the HAADF‐STEM image along PMN‐PT[01$\bar{1}$] zone axis. Although an unclear interface partially exists regarding the HAADF‐STEM image along PMN‐PT[100], a layered structure of Co_3_Mn and Fe/V lattices on PMN‐PT is observed. From these images, we infer that (001)‐oriented V layer is epitaxially grown on the PMN‐PT(011), leading to the highly (211)‐oriented Fe and Co_3_Mn layers on PMN‐PT(011). In addition, the (211)‐oriented Co_3_Mn film grown on PMN‐PT(011) exhibits two distinct epitaxial relationships related by a 180° in‐plane rotation

Co_3_Mn(211)[1$\bar{1}$ $\bar{1}]||\mathrm{PMN}$‐PT(011)[01$\bar{1}$] (variant A) and Co_3_Mn(211)[$\bar{1}11]||\mathrm{PMN}$‐PT(011)[01$\bar{1}$] (variant B), as schematically illustrated in Figure 3e. From these findings, we conclude that a Co_3_Mn/Fe/V/PMN‐PT multiferroic heterostructure having a controllable orbital magnetic moment mediated by strain has been demonstrated.

### 2.3 Giant CME Effect

**FIGURE 4:**
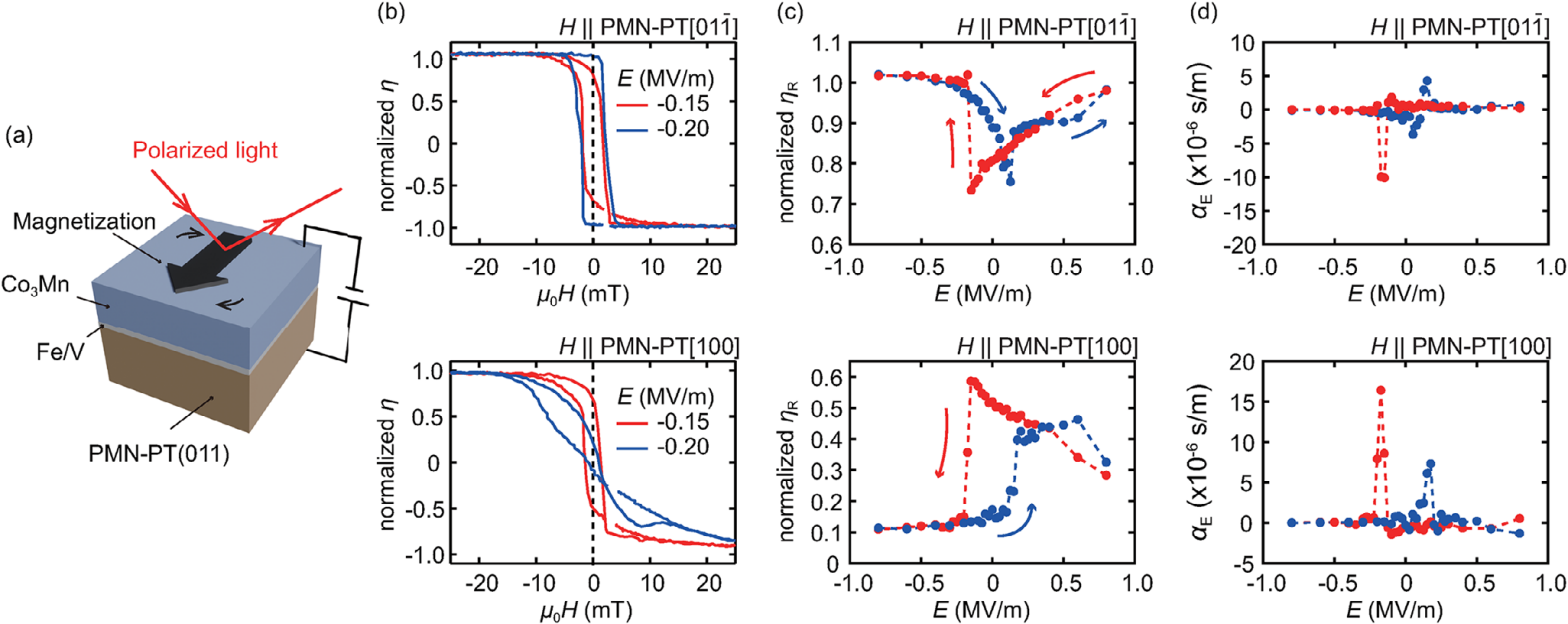
(a) Experimental setup for evaluating the converse magnetoelectric (CME) effect at room temperature. (b) Magnetic‐field dependence of the normalized magneto‐optic Kerr ellipticity (*η*) at different *E* during the down‐sweep, (c) *E* dependence of the normalized *η* at the remanent state (*η*_R_), and (d) CME coupling coefficient (*α*_E_), measured along the PMN‐PT[01$\bar{1}$] (top) and PMN‐PT[100] (bottom) directions. The blue and red plots represent the up‐sweep and down‐sweep data, respectively, within an *E* range of ±0.8 MV m^−1^.

To characterize the *E* effect on the magnetic properties of the Co_3_Mn(5)/Fe(2)/V(2)/PMN‐PT(011) heterostructure (thickness is in nm), we performed magneto‐optic Kerr ellipticity (*η*) measurements at room temperature under applying *E*. A schematic of the experimental setup is shown in Figure 4a. Figure 4b shows the normalized *η* versus external magnetic fields applied along the PMN‐PT[01$\bar{1}$] and PMN‐PT[100] directions at two different *E* values. For both magnetic field directions, the Kerr hysteresis loops clearly change upon the application of *E*, indicating the emergence of a CME effect at room temperature. To further examine the CME effect, we concentrate on the *E* dependence of the magnetic remanent state. Figure 4c shows the *E* dependence of the normalized *η* at the remanent state (*η*_R_) for the data with applied magnetic fields along the PMN‐PT[01$\bar{1}$] and PMN‐PT[100] directions, where each point is deduced from the value of *η* in the remanent state of the normalized Kerr hysteresis loops at various *E* values. For both data, a steep change of *η*_R_ is observed around *E* = −0.2 MV m^−1^.

To quantitatively evaluate the CME effect, the CME coupling coefficient (*α*_E_) is roughly estimated. Here, the value of *α*_E_ is defined as $\alpha_{E}=\left|\mu_{0}\frac{dM_{R}}{dE}\right|$ where *μ*_0_ is the permeability of vacuum, and *M*_R_ is the remanent magnetization. Also, we assume that the value of *η* at the saturated state (*η*_S_) corresponds to the value of saturation magnetization (*M*_S_ ∼1500 kA m^−1^) estimated from the VSM measurements. Based on the assumption, *M*_R_ values can be determined as *M*_R_ = *M*_S_(*η*_R_/*η*_S_) for deducing *α*_E_. Figure 4d is plot of the estimated *α*_E_. These data provide an estimated value for *α*_E_ of over 1.0× 10^−5^ s m^−1^, exceeding the reported *α*_E_ (4.7−8.3 × 10^−6^ s/m) value for the (001)‐oriented Co_3_Mn/Fe/PMN‐PT(001) multiferroic heterostructure [25, 26]. These results show that the choice of the (211) orientation of Co_3_Mn is significantly important for achieving giant CME effect, as expected in Figure 2.

**FIGURE 5:**
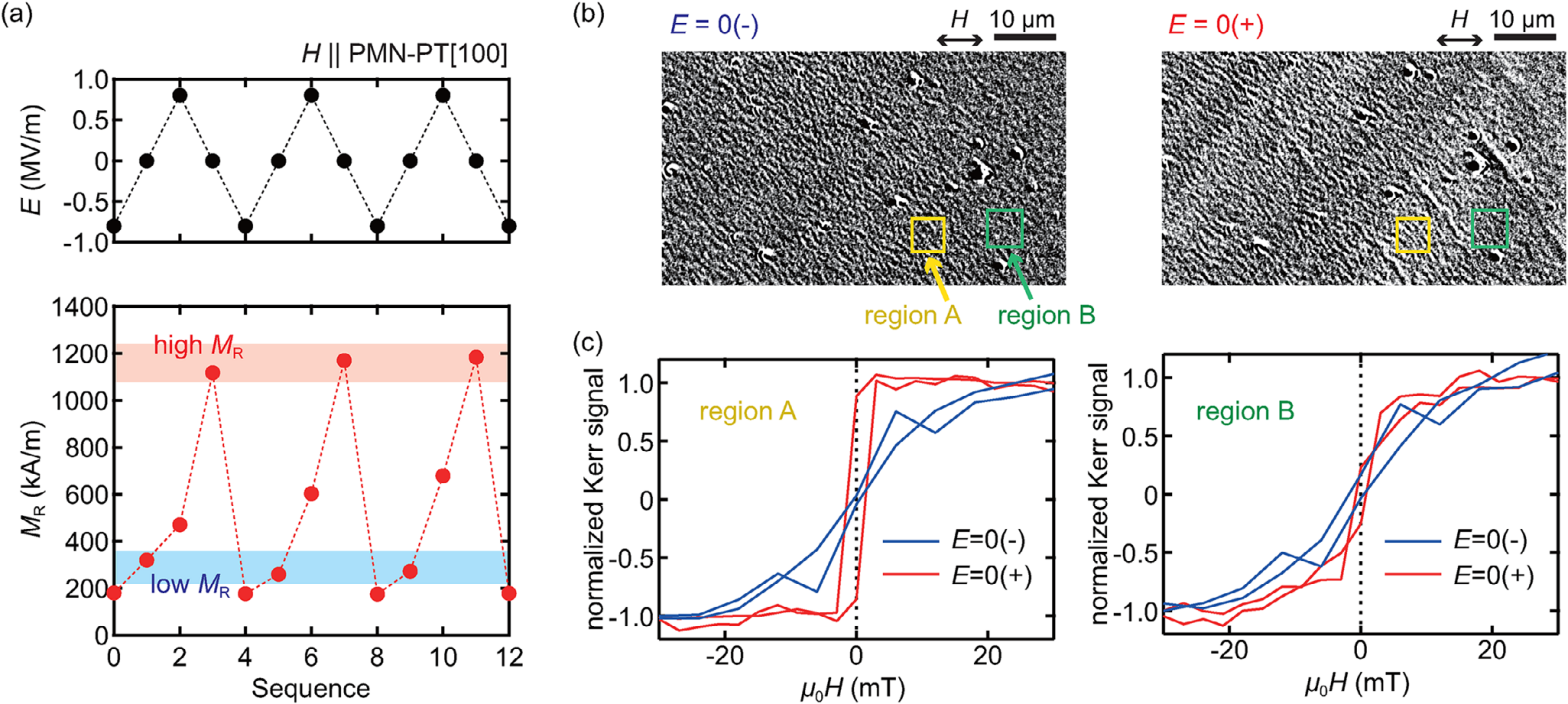
(a) Repeatable switching of the remanent magnetization (*M*_R_) at room temperature (bottom) and the corresponding change in *E* in the heterostructure (top). (b) Magnetic‐domain images in the remanent state (*H* = 0) at the *E* = 0(−) (left) and *E* = 0(+) (right) states. (c) Magnetic‐field dependence of the Kerr signal obtained from regions A and B at the *E* = 0(−) and *E* = 0(+) states, where *H* was applied along the PMN‐PT[100] direction. Regions A and B correspond to the areas indicated by the yellow and green rectangles in Figure 5b.

In addition, we examined non‐volatile binary magnetic states for evaluating an effectiveness as memory applications. As shown in Figure 4c, the magnetization directions at two different states at *E* = 0 are switched during the *E* sweeping process. We further investigated the repeatability of this switching by measuring the remanent magnetization under the sequence of *E* shown at the top of Figure 5a. The bottom of Figure 5a displays a non‐volatile switching of the remanent magnetization vector for the measurements along PMN‐PT[100] direction. Evident variations in two different remanent magnetization states with high/low *M*_R_ are repeatedly obtained. On the other hand, the Co_3_Mn/Fe/PMN‐PT(011) multiferroic heterostructure without the V layer exhibited a small difference between the high and low *M*_R_ states, as shown in Figure S2d. Therefore, the repeatable and non‐volatile magnetization vector switchings achieved by using the Fe/V layer can be utilized as part of the technology for voltage‐induced magnetization switching in storage and/or memory devices.

Finally, we discuss strategies for further enhancing the non‐volatile properties. For example, as shown in the bottom of Figure 4c, the *η*_R_ values at *E* = 0 during the down‐sweep and up‐sweep are 0.52 and 0.17, respectively. These values correspond to a change in the magnetization direction from approximately 60° to 80° with respect to the PMN‐PT[100] direction. If 90° magnetization vector switchings occurred after applying *E*, the *η*_R_ values should be 1.0 and 0 at *E* = 0 during the down‐sweep and up‐sweep, respectively. That is, the present data is insufficient to show 90° magnetization vector switchings by applying *E*, and further enhancement of the difference is desirable for a sufficient read margin for memory operation. To explore possible improvement strategies, we examine the magnetic domain structures of the grown Co_3_Mn layer. Magnetic domain images were acquired under two zero‐*E* conditions: one after applying a positive field of +0.8 MV m^−1^ and the other after applying a negative field of ‐0.8 MV m^−1^. Hereafter, these states are referred to as *E* = 0(+) and *E* = 0(−), respectively. Figure 5b presents representative magnetic domain images in the remanent magnetic state for the *E* = 0(−) (left) and *E* = 0(+) (right) states, obtained after applying *H* along the PMN‐PT[100] direction. The *E* = 0(−) state exhibits a uniform dark contrast, indicating a homogeneous magnetic domain configuration. In contrast, the *E* = 0(+) state shows the coexistence of dark and bright regions, corresponding to the formation of multi‐domain structures. These observations suggest that the *E*‐induced modulation of the magnetic state exhibits spatial dependence. To further clarify this behavior, the magnetic‐field dependence of the Kerr signal was analyzed for local regions A and B, as indicated by the yellow and green rectangles in Figure 5b. Figure 5c shows the Kerr signal as a function of the magnetic field for these regions. For each region, the signals corresponding to the *E* = 0(−) (blue) and *E* = 0(+) (red) states are plotted. In region A, the Kerr signal exhibits a pronounced change: the normalized value at the remanent state (*H* = 0) increases from 0.03 to 0.87 when switching from the *E* = 0(−) to the *E* = 0(+) state, indicating a nearly 90° rotation of the magnetic easy axis. This difference in the remanent signal is larger than that shown in the bottom of Figure 4c, where the *η*_R_ values at the *E* = 0(−) and *E* = 0(+) states are 0.17 and 0.52, respectively. In contrast, region B exhibits only a slight change in the Kerr signal, revealing that the response of the magnetic states to piezostrain is spatially inhomogeneous. Similar behavior is also observed in other regions (not shown). We presume that this behavior originates from the intrinsic strain inhomogeneity of relaxor ferroelectrics such as PMN‐PT, which arises from the coexistence of polar nanoregions and ferroelectric domains [27]. Since it has been shown that the magnitude of *K*_g_ significantly influences the CME effect [17], such intrinsic inhomogeneity in the ferroelectric substrate is expected to induce a spatial variation of *K*_g_, thereby leading to a spatially nonuniform electric‐field response of the magnetic anisotropy.

The domain‐imaging results highlight the importance of controlling the microscopic magnetic domain configuration. In particular, suppressing spatial inhomogeneity is essential for achieving nonvolatility. To address the above issues, an effective approach would be to modulate the magnetic easy axis of the Co_3_Mn layer by optimizing the shape anisotropy through micro‐patterning or by fabricating in‐plane electrodes to generate a more uniform piezoelectric strain [28]. These strategies would not only ensure reliable device operation but also enable a more precise evaluation of the CME effect, consistent with predictions based on the controllability of the orbital magnetic moments.

Finally, we briefly comment on the contribution of the spin magnetic moment (*m*_s_) to *E*_MCA_. In addition to the orbital contribution, possible effects arising from changes in *m*_s_ also exist. To clearly distinguish the orbital and spin contributions [16], further studies using experimental techniques such as XMCD measurements under an *E* would be valuable. The present study demonstrates that enhancement of the CME effect is achieved mainly by growing a (211)‐oriented Co_3_Mn layer with a strong strain dependence of *m*_orb_. Our results indicate that the strain‐induced modulation of *E*_MCA_ in Co_3_Mn is closely related to the anisotropy of the orbital magnetic moment, suggesting that control of orbital magnetic moments can be an effective strategy for achieving a giant CME effect in multiferroic heterostructures.

## 3 Conclusions

We have proposed and demonstrated a material design strategy based on utilizing the control of orbital magnetic moments for multiferroic heterostructures with the giant CME effect. In this study, we focused on a heterostructure composed of metastable bcc Co_3_Mn and PMN‐PT(011). By inserting an appropriate Fe/V layer between Co_3_Mn and PMN‐PT(011), we experimentally obtained the highly (211)‐oriented bcc Co_3_Mn layer with an effective large orbital magnetic moment change. As a result, we experimentally showed the giant CME effect with repeatable and nonvolatile magnetization vector switching. Our proposal is one of the important strategies toward the development of room‐temperature electric‐field‐controlled spintronic devices with ultra‐low power consumption.

## 4 Methods

### 4.1 Computational Details in DFT Calculations

First‐principles calculations were performed on the basis of density functional theory (DFT) within the generalized gradient approximation [29]. Inner core electrons were treated as frozen using the projector‐augmented wave (PAW) method as implemented in the VASP code [30, 31, 32]. As for convergence criteria, the maximum force on each atom and the unit cell was 0.02 eV Å^−1^, while the total‐energy variation was set as approximately 10^−6^ eV that was tighten to 5 × 10^−7^ eV in evaluating the MCA. The cutoff energy was set as 351 eV for structural optimization, while it was decreased to 270 eV in evaluating the MCA. In the sampling of single‐electron states, *k*‐grid spacing was set as approximately $0.1\times 0.1\times 0.1{Å}^{-3}$. We evaluated the magnetocrystalline anisotropy energy by including the spin‐orbit coupling explicitly in the Kohn‐Sham Hamiltonian of DFT. For convenience, the bcc‐based *D*0_3_ structure was used for the bcc phase.

Here, two unit cells were used to calculate (211)‐oriented and (001)‐oriented Co_3_Mn. In the case of the (001) plane, the conventional cubic unit cell was used with the *a* vector in the [100] direction and the *b* vector in the [010] direction. In the case of the (211) plane, the orthorhombic unit cell was defined with the *a* vector in the [0$\bar{1}$1] direction and the *b* vector in the [1$\bar{1}\bar{1}$] direction. For each case, the lattice constant *a* was strained from its equilibrium value *a*_0_, while the *b* direction was deformed from the equilibrium lattice constant *b*_0_ with the constraint *ab* = *a*_0_ *b*_0_. *E*_MCA_ was defined as *E* ^*b*^ − *E* ^*a*^, where *E* ^*a*^ (*E* ^*b*^) is the total energy per atom with the magnetization in the *a* (*b*) direction. Δ*m*_orb_ was defined as $m_{\mathrm{orb}}^{b}-m_{\mathrm{orb}}^{a}$, where the orbital moments $m_{\mathrm{orb}}^{a}$ and $m_{\mathrm{orb}}^{b}$ were defined in the same fashion and averaged per atom.

### 4.2 Film Growth and Characterization

The Co_3_Mn thin films were grown on 0.5‐mm‐thick PMN‐PT(011) substrates using an MBE technique, where the composition of PMN‐PT was Pb(Mg_1/3_Nb_2/3_)_(1 − *x*)_O_3_‐PbTi_*x*_O_3_ (*x* = 0.29 − 0.32), close to the morphotropic phase boundary showing large piezoelectricity [33]. Flat and clean substrate surfaces were obtained by heat treatment (400°C for 20 min) prior to film growth, where the heat‐treatment temperature was sufficiently lower than that in the literature (∼800°C) showing a giant CME effect [34]. This ensures that any loss of piezoelectricity would be negligible. After cooling the substrate to 300°C, Fe/V underlayers were grown. Subsequently, a Co_3_Mn film with various thicknesses ($t_{{\mathrm{Co}}_{3}\mathrm{Mn}}$) was grown under 100°C by co‐evaporation using Knudsen cells [25, 26]. After growth, the Co_3_Mn/PMN‐PT(011) multiferroic heterostructures were characterized. First, the heterostructures were evaluated by X‐ray diffraction (XRD) (Rigaku SmartLab) for out‐of‐plane analyses. High‐resolution transmission electron microscopy (HRTEM) was also performed, and the specimen preparation for TEM analysis was conducted using focused ion beam techniques.

To measure the conventional magnetic properties of the grown multiferroic heterostructures, we used a vibrating sample magnetometer (VSM) at room temperature. The CME effect was characterized by longitudinal magneto‐optic Kerr effect measurements with the application of an *E*. We used a magneto‐optic Kerr‐effect measurement system with an LED light source. Here, the wavelength was 670 nm, the beam size was about *ϕ* 0.7 mm, and the incident angle was about 25°.

To apply an *E* to the PMN‐PT substrate along the [011] direction, a Au(100 nm)/Ti(3 nm) electrode was deposited on the backside of the PMN‐PT substrate, where the Co_3_Mn film was also utilized as a top electrode. Prior to evaluating the CME effect, an *E* of ‐0.8 MV m^−1^ was first applied. Then, the amplitude of *E* was gradually changed from ‐0.8 MV m^−1^ to +0.8 MV m^−1^, then back to *E* = −0.8 MV m^−1^. Here, *E* of 0.8 MV m^−1^ corresponds to a voltage of 400 V, since the thickness of the PMN‐PT substrate is 0.5 mm. At each step, the Kerr‐ellipticity magnitude was obtained by measuring the hysteresis loops as a function of *H* along the PMN‐PT[01$\bar{1}$] or [100] direction.

## Conflicts of Interest

The authors declare no conflicts of interest.

## Supporting information


**Supporting File**: advs73972‐sup‐0001‐SuppMat.pdf.

## Data Availability

The data that support the findings of this study are available from the corresponding author upon reasonable request.
