# Supplementary material for: Orbital Magnetic Moment Controlled Converse Magnetoelectric Effect in bcc‐Co3Mn/Fe/V/PMN‐PT Multiferroic Heterostructures
Source: Adv Sci (Weinh). 2026 Jan 27:e22581. Online ahead of print. doi: 10.1002/advs.202522581 (PMC13325553; doi:10.1002/advs.202522581)
Supplement: Supplementary file 1 — Supporting File: advs73972‐sup‐0001‐SuppMat.pdf. [file ADVS-9999-e22581-s001.pdf]

Supplementary information

Supplementary information 1

Figure S1 displays wide range X-ray diffraction (XRD) $\omega - 2\theta$ profiles for the Co_3Mn/Fe/V/PMN-PT(011) heterostructure (red), Co_3Mn/Fe/PMN-PT(011) heterostructure (blue), and Co_3Mn/V/PMN-PT(011) heterostructure(black). The peaks denoted by the black diamond and asterisks originate from the Co_3Mn(211) and PMN-PT substrate, respectively. Only the Co_3Mn 211 diffraction peak is detected, and other diffraction peaks from Co_3Mn are not observed for the heterostructures using Fe(2)/V(2) and Fe(2) buffer layers.

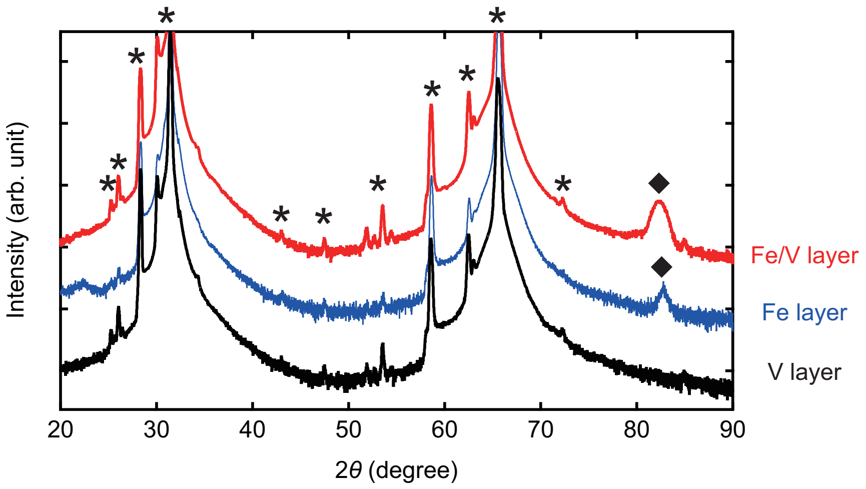

Figure S1 Wide range X-ray diffraction (XRD) $\omega - 2\theta$ profiles for the Co_3Mn/Fe/V/PMN-PT(011) heterostructure (red), Co_3Mn/Fe/PMN-PT(011) heterostructure (blue), and Co_3Mn/V/PMN-PT(011) heterostructure(black). The peaks denoted by the black diamond and asterisks originate from the Co_3Mn(211) and PMN-PT substrate, respectively.

Supplementary information 2

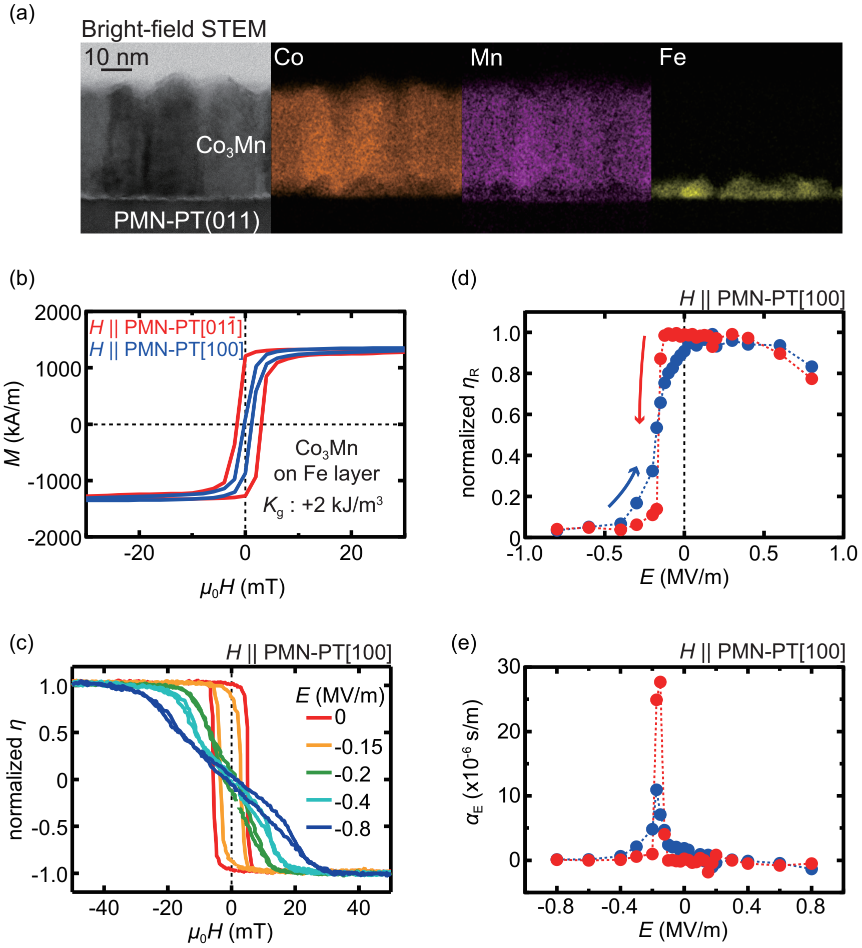

Figure S2 (a) Bright-field cross-sectional STEM images and EDX elemental maps of Co_3Mn/Fe/PMN-PT(011) heterostructure with zone axes of PMN-PT[100]. (b) Magnetization curves measured at room temperature under the magnetic fields along the PMN-PT[01$\bar{1}$] (red) and PMN-PT[100] (blue) directions. (c) The magnetic-field dependence of the normalized MOKE ellipticity (η) at different E along the PMN-PT[100]. (d) E dependence of the normalized η at the remanent state (η_R) along the PMN-PT[100]. The blue and red plots present the up-sweep and down-sweep data, respectively, within an E of ±0.8 MV/m. (e) Plots of the CME coupling coefficient (α_E) as a function of E, where the data is estimated from the data in Figure S2(d).

Figure S2(a) displays the bright-field cross-sectional STEM images and energy-dispersive X-ray spectroscopy (EDX) elemental maps of the Co_3Mn/Fe/PMN-PT(011) heterostructure. The Fe layer exhibits relatively large roughness compared to the heterostructure with the Fe/V insertion layer shown in Figure 3(c). This result highlights the importance of the Fe/V insertion layer in achieving a smoother Co_3Mn layer. Figure S2(b) shows the magnetization curves measured at room temperature under the magnetic fields along the PMN-PT[01$\bar{1}$] (red) and PMN-PT[100] (blue) directions. The value of K_g estimated from Equation (2) is 2 kJ/m^3, which is smaller than that for the case with the Fe/V layer insertion. Figure S2(c) shows the magnetic-field dependence of the normalized MOKE ellipticity (η) at different E along the PMN-PT[100]. The Kerr hysteresis loops change upon the application of E. Figure S2(d) shows E dependence of the normalized η at the remanent state (η_R) along the PMN-PT[100], and Figure S2(e) displays the plots of the CME coupling coefficient (α_E) as a function of E, where the data is estimated from the data in Figure S2(d). The blue and red plots present the up-sweep and down-sweep data, respectively, within an E of ±0.8 MV/m. The data provides an estimated value for α_E of over 1.0×10^{-5} s/m.

Supplementary information 3

Figure S3(a) shows the magnetization curves for the Co_3Mn/V/PMN-PT(011) heterostructure at room temperature, where the magnetic fields are applied along the PMN-PT[01$\bar{1}$] (red) and PMN-PT[100] (blue) directions. The magnetic easy axis is found to emerge along the PMN-PT[100] directions. This easy-axis direction is rotated by 90 degrees compared with that of the Co_3Mn/Fe/V/PMN-PT(011) and Co_3Mn/Fe/PMN-PT(011) heterostructures.

Figure S3(b) shows E dependence of the normalized η at the remanent state (η_R) along the PMN-PT[100]. The blue and red plots present the up-sweep and down-sweep data, respectively, within an E of ± 0.8 MV/m. No distinct change in η_R is observed by applying an electric field. Thus, we infer that a clear difference in the sign of K_g affects the appearance of CME effect. Specifically, the Co_3Mn/V/PMN-PT heterostructure without the CME effect has a negative K_g, whereas the Co_3Mn/[(Fe/V) or Fe]/PMN-PT structures with the CME effect shows a positive value. However, the possibility that the presence of the bottom Fe layer modulates the anisotropy constant cannot be excluded, and a detailed study is required to clarify this effect.

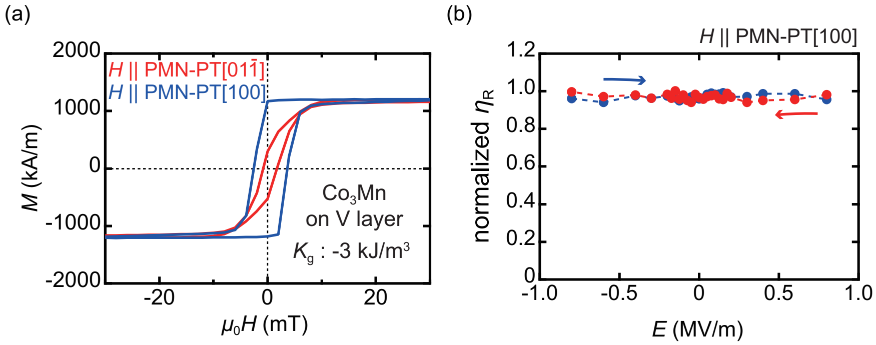

Figure S3 (a) Magnetization curves for the Co_3Mn with the V layer, where the external magnetic fields (H) are applied along the PMN-PT[01$\bar{1}$] (red) and PMN-PT[100] (blue) crystallographic directions. (b) E dependence of the normalized η at the remanent state (η_R) along the PMN-PT[100]. The blue and red plots present the up-sweep and down-sweep data, respectively, within an E of ± 0.8 MV/m.
